# Identification of microRNA differentially expressed in three subtypes of non-small cell lung cancer and in silico functional analysis

**DOI:** 10.18632/oncotarget.20218

**Published:** 2017-08-12

**Authors:** Yanjun Hu, Luqing Wang, Jingxian Gu, Kai Qu, Yunxia Wang

**Affiliations:** ^1^ Department of Clinical Laboratory, Liaocheng People’s Hospital, Taishan Medical College, Liaocheng 252000, Shandong Province, China; ^2^ Department of Nuclear Medicine, Radioimmunology Room, Liaocheng People’s Hospital, Taishan Medical College, Liaocheng 252000, Shandong Province, China; ^3^ Department of Hepatobiliary Surgery, The First Affiliated Hospital of Xi’an Jiaotong University, Xi’an 710061, China; ^4^ Department of Intensive Care Unit, Liaocheng Infectious Diseases Hospital, Liaocheng 252000, Shandong Province, China

**Keywords:** non-small cell lung cancer, histological subtype, differentially expressed miRNAs

## Abstract

Emerging studies demonstrated that miRNAs played fundamental roles in lung cancer. In this study, we attempted to explore the clinical significance of the miRNA signature in different histological subtypes of non-small cell lung cancer (NSCLC). Three miRNome profiling datasets (GSE19945, GSE25508 and GSE51853) containing lung squamous cell carcinoma (SCC), lung adenocarcinoma (ADC) and large cell lung cancer (LCLC) samples were obtained for bioinformatics and survival analysis. Moreover, pathway enrichment and coexpression network were performed to explore underlying molecular mechanism. MicroRNA-375 (miR-375), miR-203 and miR-205 were identified as differentially expressed miRNAs (DEmiRNAs) which distinguished SCC from other NSCLC subtypes. Pathway enrichment analysis suggested that Hippo signaling pathway was combinatorically affected by above mentioned three miRNAs. Coexpression analysis of three miRNAs and the Hippo signaling pathway related genes were conducted based on another dataset, GSE51852. Four hub genes (*TP63*, *RERE*, *TJP1* and *YWHAE*) were identified as the candidate targets of three miRNAs, and three of them (*TP63*, *TJP1* and *YWHAE*) were validated to be downregulated by miR-203 and miR-375, respectively. Finally, survival analysis further suggested the prognostic value of three-miRNA signature in SCC patients. Taken together, our study compared the miRNA profiles among three histological subtypes of NSCLC, and suggested that a three-miRNA signature might be potential diagnostic and prognostic biomarkers for SCC patients.

## INTRODUCTION

Lung cancer remains the leading cause of cancer-related death both worldwide and in China [1, 2]. Non-small cell lung cancer (NSCLC) including three main histological subtypes, lung squamous cell carcinoma (SCC), lung adenocarcinoma (ADC) and large cell lung cancer (LCLC), accounts for over 80% cases of lung cancer [3]. Increasing studies demonstrated that therapeutic effect varied in different histological subtypes of NSCLC, suggesting that early confirmation of histology is of critical importance [4-6]. Nevertheless, there are emerging researches into the biological features of different histological subtypes of NSCLC, but the fundamental molecular mechanisms remain elusive. For instance, smoking is a much greater risk of SCC than of ADC [7]. Thus, a clear understanding of the differences from morphological to genetic levels between them will definitely have a positive effect on precise therapy.

MicroRNAs (miRNAs), comprising 19-23 nucleotides or so, are a class of endogenously expressed RNAs belonging to non-coding RNA family [8]. In the last decade, miRNAs have attracted so much attention as biomarkers with great potential in the early diagnosis, and clinical outcome prediction of multiple malignancies [9-11]. In NSCLC, emerging evidence suggested miRNAs as potential diagnostic and prognostic biomarkers [12-14]. Several miRNAs were reported to be capable of distinguishing different subtypes of NSCLC [15, 16]. Therefore, exploration of miRNA-based biomarkers provides opportunities for investigate the molecular changes among different histological subtypes of NSCLC.

Recently, high-throughput platforms have been utilized as a fundamental method dealing with massive genetic data. Data from high-throughput platforms including microarrays and next-generation sequence, if analyzed by bioinformatics methods, can provide amounts of useful information for screening cancer biomarkers and therapeutic targets [17]. Gene Expression Omnibus (GEO) database (https://www.ncbi.nlm.nih.gov/geo/), hosted by the National Center for Biotechnology Information (NCBI), serves as a public genetic expression profile repository for a wide range of high-throughput experimental data. Therefore, in the present study, we adopted high-throughput data from GEO database for bioinformatics analysis to identify the differentially diagnostic miRNA signature between three subtypes of NSCLC.

## RESULTS

### Identification of differentially expressed miRNAs (DEmiRNAs) in NSCLC

**Figure 1:**
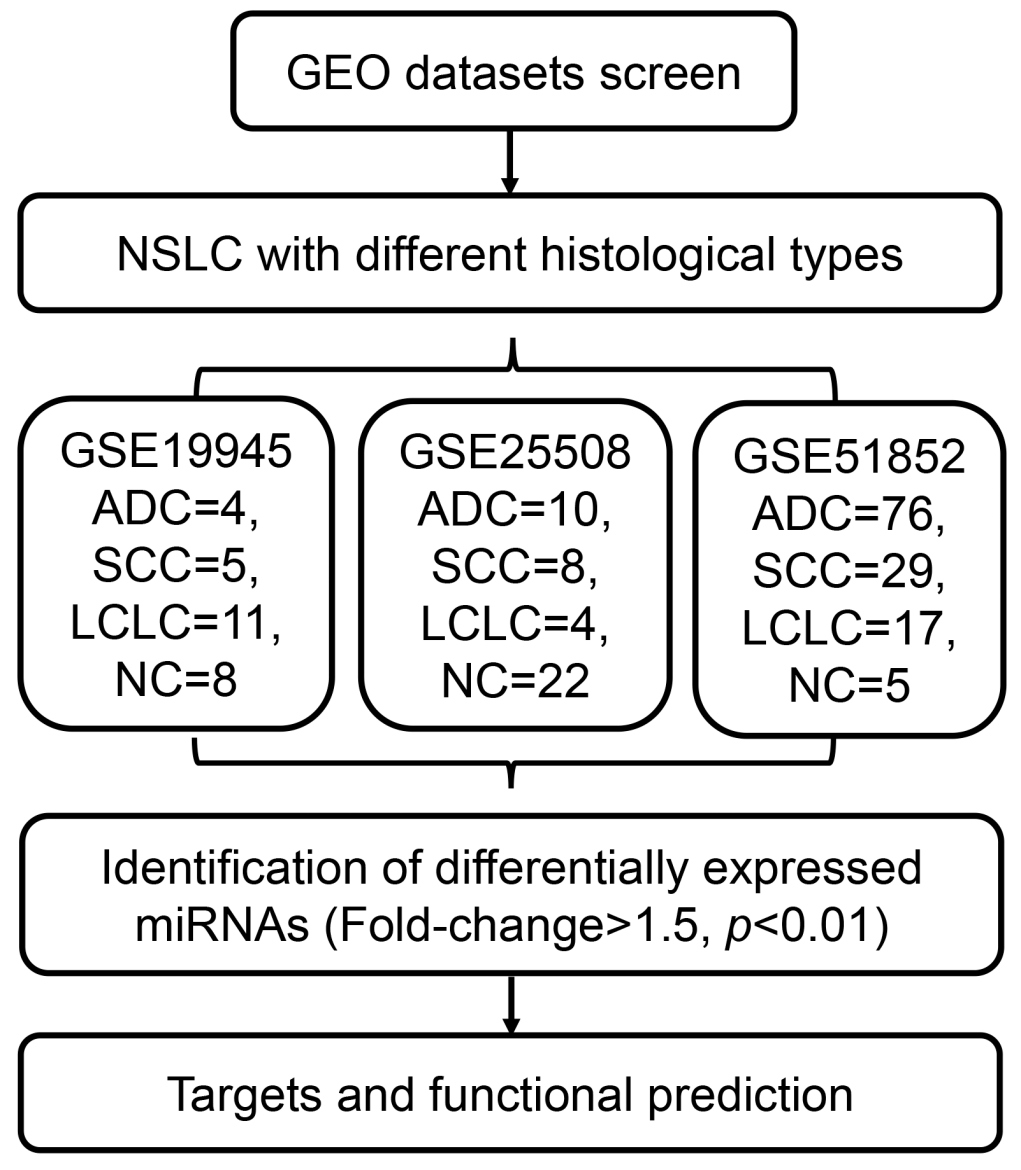
Schematic of workflow depicting the eligible datasets and analysis procedure

**Figure 2:**
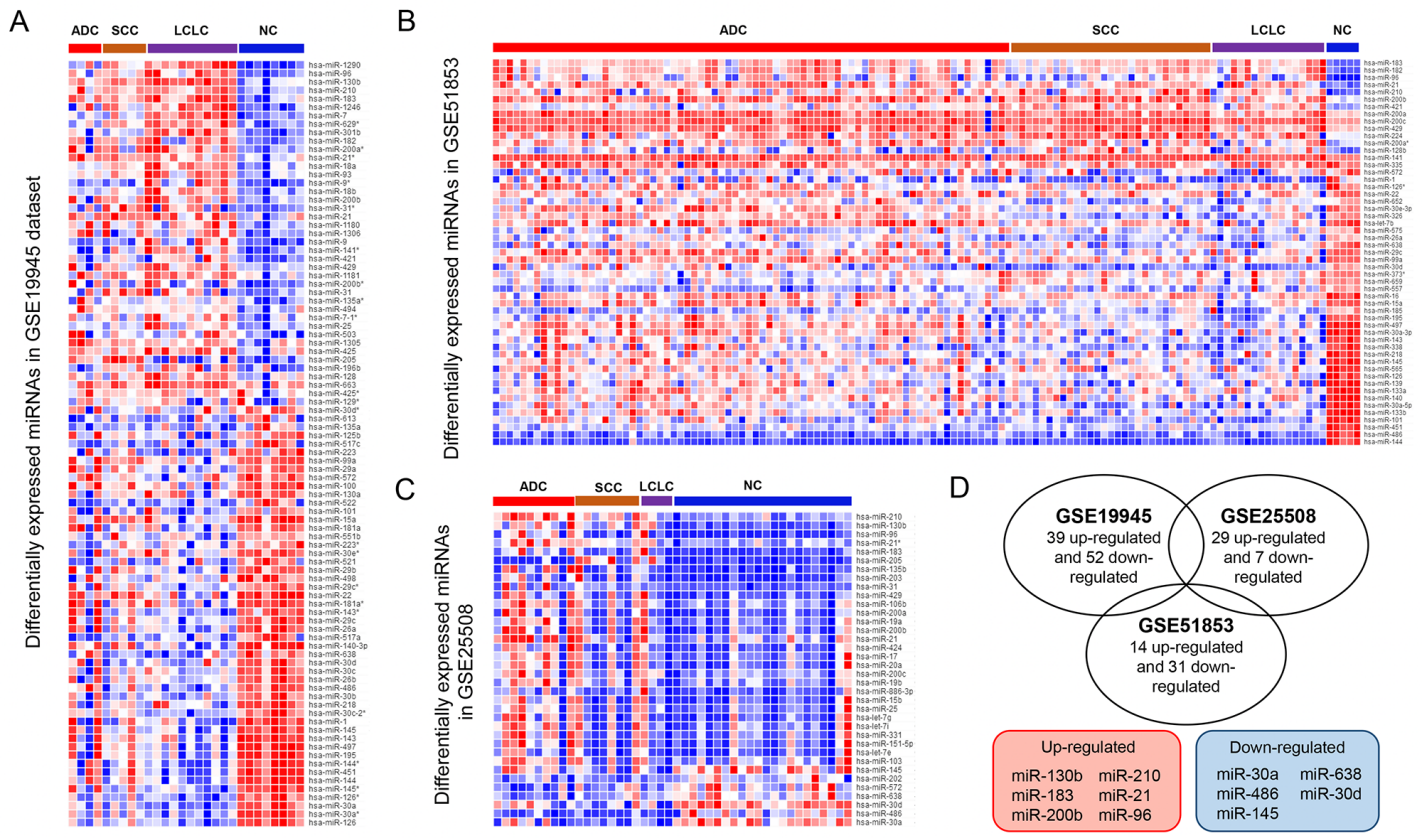
Identification of common DEmiRNAs in NSCLC Heat map of the significantly dysregulated miRNAs between the cancer and normal tissue samples in GSE19945 **(A)**, GSE51852 **(B)** and GSE25508 **(C)**. **(D)** (*Upper*) Venn diagram between the up-regulated and down-regulated miRNAs from each dataset. (*Lower*) the lists of the common dysregulated miRNAs. Red: up-regulation; blue: down-regulation.

The workflow of this study was presented in Figure 1. Firstly, we compared the miRNA expression profiles in three miRNA datasets (GSE19945, GSE25508 and GSE51852). DEmiRNAs between cancer and normal tissue samples were identified in each dataset. In GSE19945, 39 up-regulated and 52 down-regulated miRNAs were obtained (Figure 2A). Based on GSE51853, a total of 53 dysregulated miRNAs were identified including 14 up-regulated and 31 down-regulated miRNAs (Figure 2B). In GSE25508, there were 29 up-regulated and 7 down-regulated miRNAs (Figure 2C). When comparing the list of dysregulated miRNAs among three datasets, six upregulated miRNAs (miR-130b, miR-210, miR-183, miR-21, miR-200b and miR-96) and five down-regulated miRNAs (miR-30a, miR-638, miR-486, miR-30d and miR-145) (Figure 2D).

### Identification of DEmiRNAs in different histological subtypes of NSCLC

**Figure 3:**
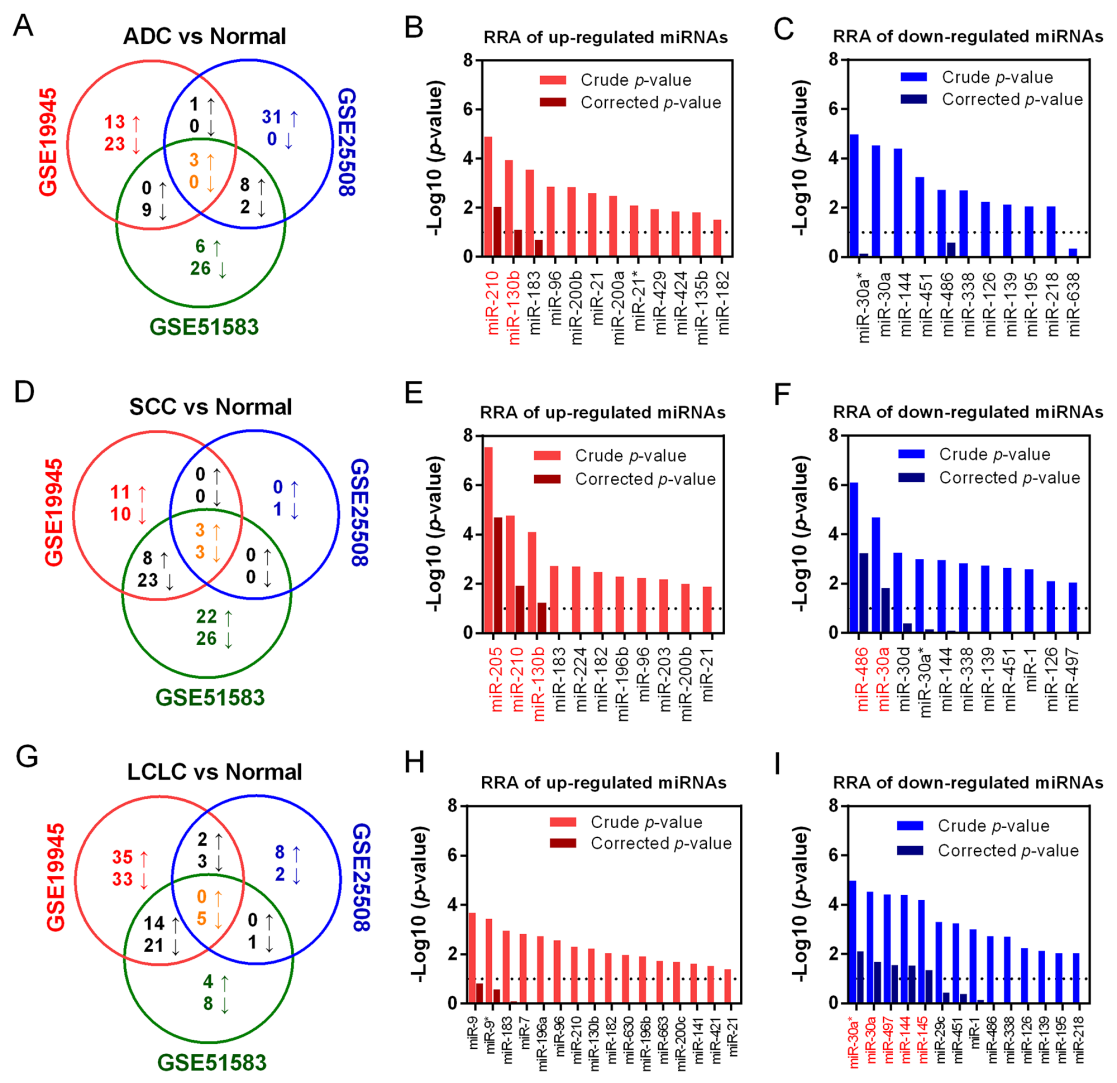
Identification of DEmiRNAs in three histological subtype of NSCLC **(A)** Overlapped DEmiRNAs in ADC were presented as Venn diagram. RRA analysis of up-regulated **(B)** and down-regulated miRNAs **(C)** to identify common DEmiRNAs in ADC. **(D)** Overlapped DEmiRNAs in SCC were presented. RRA analysis of up-regulated **(E)** and down-regulated miRNAs **(F)** in SCC were listed. **(G)** Overlapped DEmiRNAs in LCLC were presented. RRA analysis of up-regulated **(H)** and down-regulated miRNAs **(I)** in LCLC were listed. For the RRA column diagram, red: crude p-value (up-regulation); Dark red: corrected p-value (up-regulation). Blue: crude p-value (down-regulation); Dark blue: corrected p-value (down-regulation).

We further compared miRNA expression profiles between cancer and normal tissue samples in different histological subtypes of NSCLC (ADC, SCC and LCLC) of each dataset. A Venn diagram was conducted to examine the overlap of resulting DEmiRNAs lists from three datasets in each histological type of NSCLC. In ADC, there were 12 up- and 11 down-regulated miRNAs listed in more than two datasets (Figure 3A). Robust rank aggregation (RRA) analysis showed miR-210 and miR-130b was significantly up-regulated with a Bonferroni-corrected *p*-value less than 0.05 (Figure 3B), but no downregulated miRNA reached statistical significance after Bonferroni correction (Figure 3C). Similarly, a total of 11 up- and 26 down-regulated miRNAs commonly listed in SCC datasets (Figure 3D). Integrated meta-analysis confirmed that miR-205, miR-210 and miR-130b was up-regulated, and miR-486 and miR-30a were down-regulated (Figure 3E and 3F). In LCLC, there were 14 up- and 27 down-regulated miRNAs were selected (Figure 3G), and miR-30a*, miR-30a, miR-497, miR-144 and miR-145 were identified as commonly down-regulated miRNAs in LCLC (Figure 3H and 3I).

### MiR-203, miR-205 and miR-375 distinguished SCC from other NSCLC subtypes

**Figure 4:**
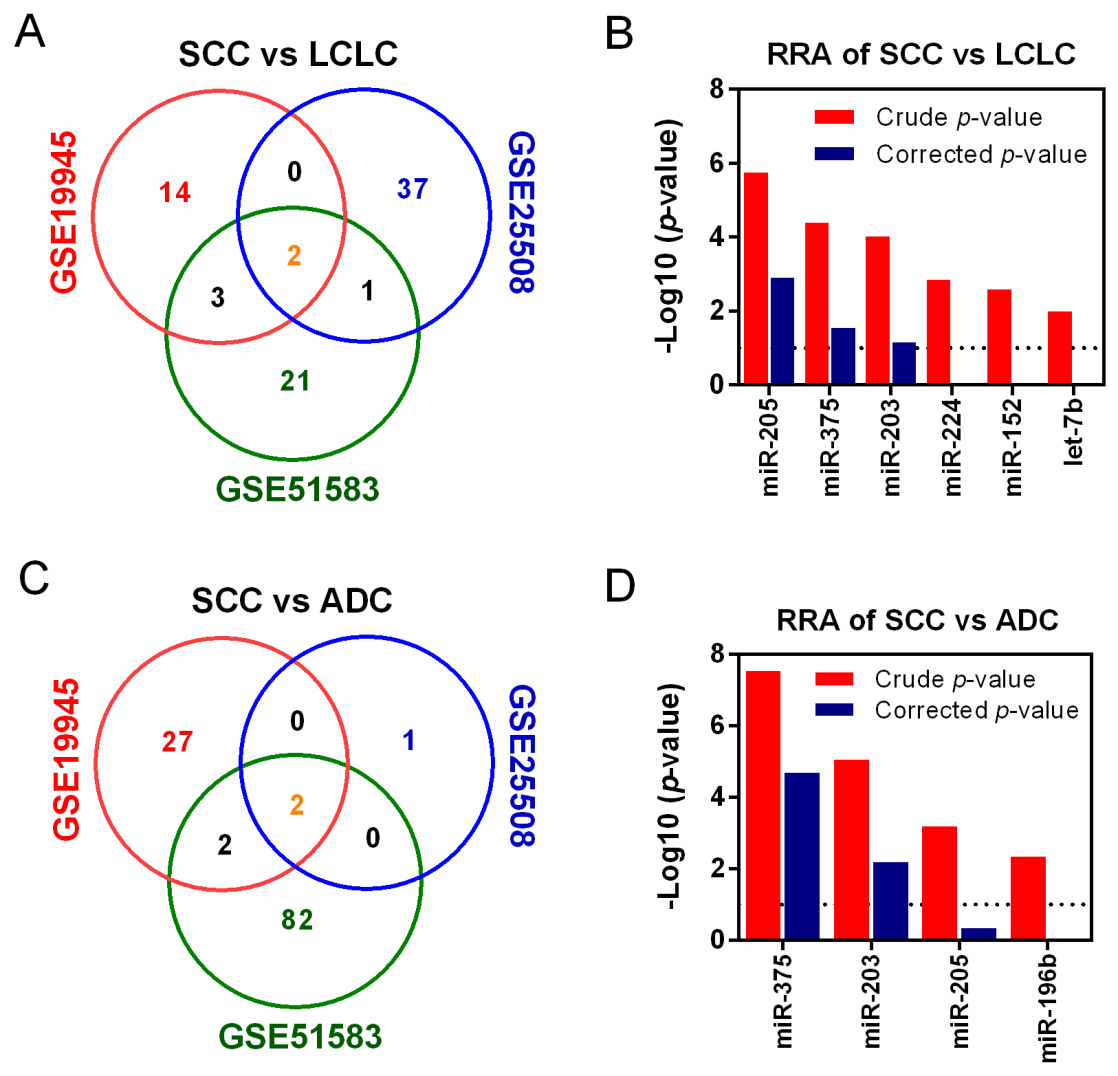
Identification of DEmiRNAs among histological subtypes of NSCLC Venn diagram of overlapped dysregulated miRNAs of SCC vs. LCLC **(A)** and SCC vs. ADC **(C)**. Common DEmiRNAs of SCC vs. LCLC **(B)** and SCC vs. ADC **(D)** were identified using RRA method. For the RRA column diagram, red: rude p-value; blue: corrected p-value.

We also explored the DEmiRNAs between SCC and other two histological subtypes of NSCLC, LCLC and ADC. It was found that a total of 6 miRNAs were significantly dysregulated from the comparison of SCC versus LCLC (Figure 4A). The corresponding RRA approach revealed that miR-205, miR-375 and miR-203 were the commonly dysregulated miRNAs (Figure 4B). When comparing SCC with ADC, four significantly dysregulated miRNAs were selected (Figure 4C). The comprehensive meta-analysis showed that miR-375 and miR-203 were significantly dysregulated (Figure 4D). Although miR-205 was not significant, it was the third dysregulated miRNA collectively. In addition, we did not obtain positive result from comparison between ADC and LCLC (data not shown). Interestingly, miR-375, miR-203 and miR-205 were listed in both SCC versus ADC and SCC versus LCLC. Therefore, miR-375, miR-203 and miR-205 were selected for further analysis.

### Function prediction of miR-203, miR-205 and miR-375 in SCC

**Figure 5:**
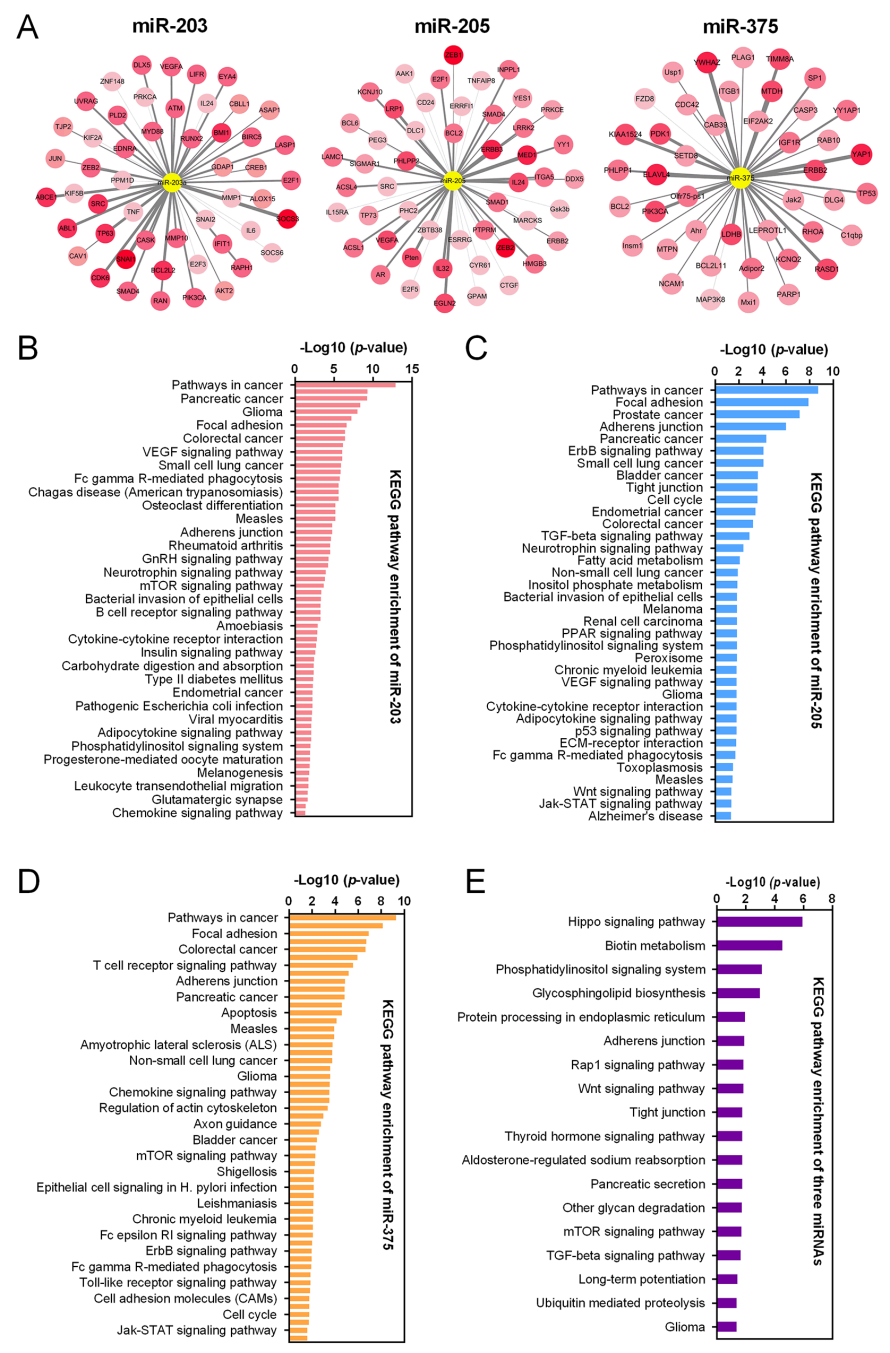
MiRNA-gene network and KEGG pathway enrichment **(A)** The interaction between three miRNAs (miR-203, miR-205 and miR-375) and of their predicted target genes. The central yellow circles represented miRNAs. The color of the circles with the names of the target genes on it varies according to their cumulative weighted context++ score. KEGG pathway enrichments of predicted target genes of miR-203 **(B)**, miR-205 **(C)** and miR-375 **(D)**, were presented, respectively. **(E)** The combinatorial effects of three miRNAs in KEGG pathways were predicted.

**Figure 6:**
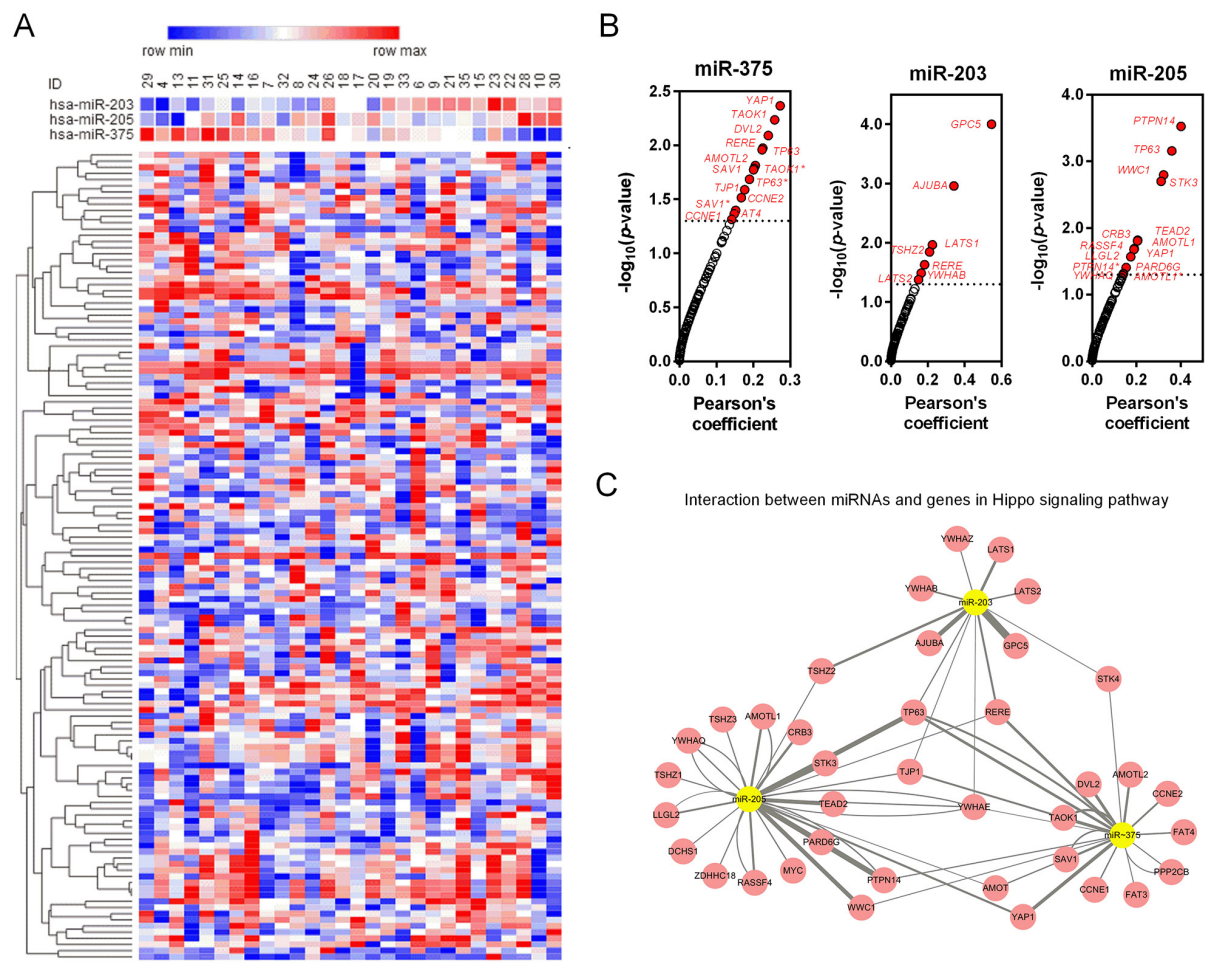
Coexpression analysis of three miRNAs and Hippo signaling related genes **(A)** The expression profile of 3 miRNAs (miR-203, miR-205 and miR-375) and 90 Hippo signaling related genes was presented as heatmap. **(B)** Pearson correlation for each miRNA and 90 Hippo signaling related genes. The genes which considered to have statistically significant relationship with the miRNA were marked red. **(C)** The interaction network of the significant couples derived from Pearson correction.

**Figure 7:**
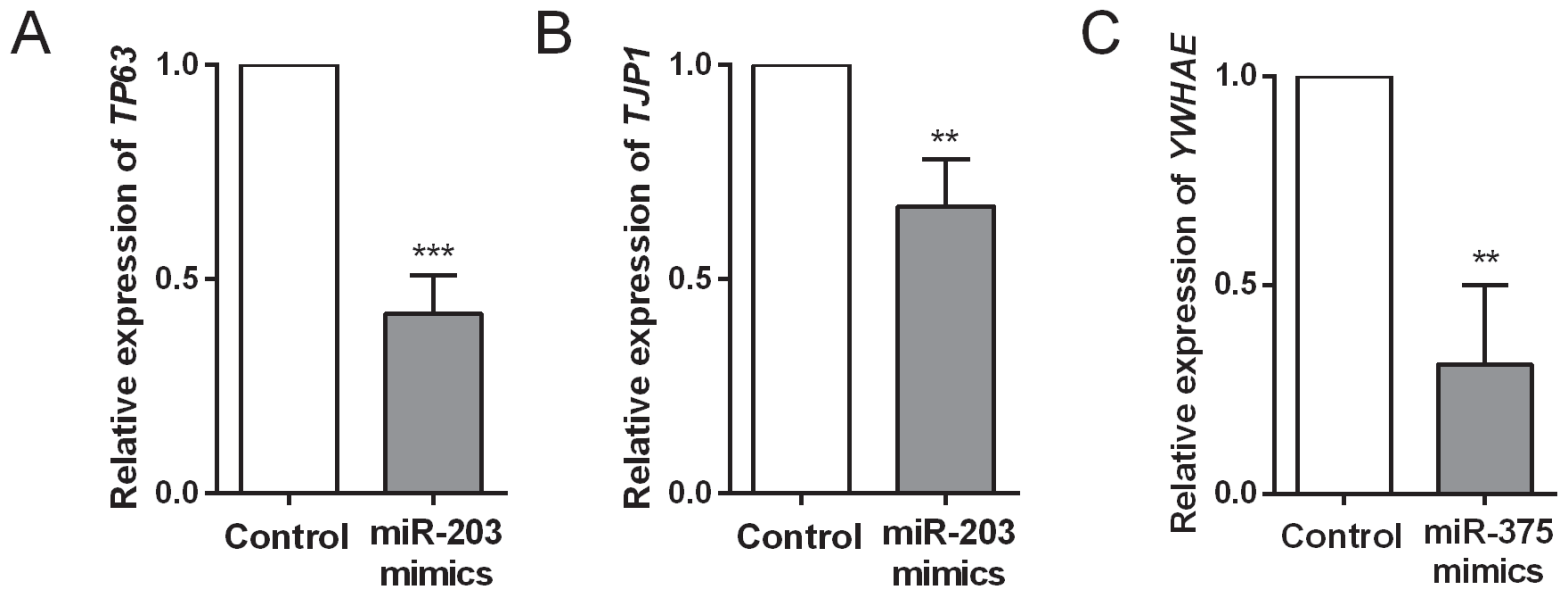
Comparison of target gene expression levels between control and miRNA mimics -transfected HCC cells **(A)** Downregulation of *TP63* by miR-203 mimics transfection; **(B)** Downregulation of *TJP1* by miR-203 mimics transfection; **(C)** Downregulation of *YWHAE* by miR-375 mimics transfection.

Target genes of miR-203, miR-205 and miR-375 were predicted by TargetScan and mapped in Cytoscape (Figure 5A). Kyoto Encyclopedia of Genes and Genomes (KEGG) pathway enrichments of target genes were carried out, respectively (Figure 5B-5D). “Pathway in cancer (KEGG_05200)”, “Focal adhesion (KEGG_04510)” and “Adherens junction (KEGG_04520)” were commonly top pathways by function prediction of three miRNAs. Moreover, the combinatorial effects of three miRNAs in pathways were predicted by the DIANA tools (http://www.microrna.gr/miRPathv2). Hippo signaling pathway was the most enriched KEGG pathway (Figure 5E). Recently, increasing evidence suggested that Hippo pathway, which was demonstrated to regulate cell proliferation, differentiation and migration, played a critical role in lung cancer, particularly in NSCLC [23-25]. We therefore employed another GEO dataset, GSE51852, to further analysis the expression correlations between three miRNAs and genes in Hippo signaling pathway (Figure 6A). Pearson correlation was calculated and yielded three sets of genes that were significantly correlated with miR-375, miR-203 and miR-205 (Figure 6B). We then conducted interaction network based on three miRNAs and their targets which reached statistical significance. From the comprehensive network, 4 genes (*TP63*, *RERE*, *TJP1* and *YWHAE*) were found to be interacted with all three miRNAs, and 6 genes (*YAP1*, *AMOT*, *WWC1*, *PTPN14*, *TSHZ2* and *STK4*) were interacted with two miRNAs (Figure 6C). In addition, we also validated the regulationship between three miRNAs and above Hippo signaling genes. Our data revealed that *TP63* and *TJP1* were downregulated by miR-203 (Figure 7A and 7B), meanwhile, *YWHAE* was downregulated by miR-375 (Figure 7C).

### Prognostic values of miR-203, miR-205 and miR-375 in SCC

**Figure 8:**
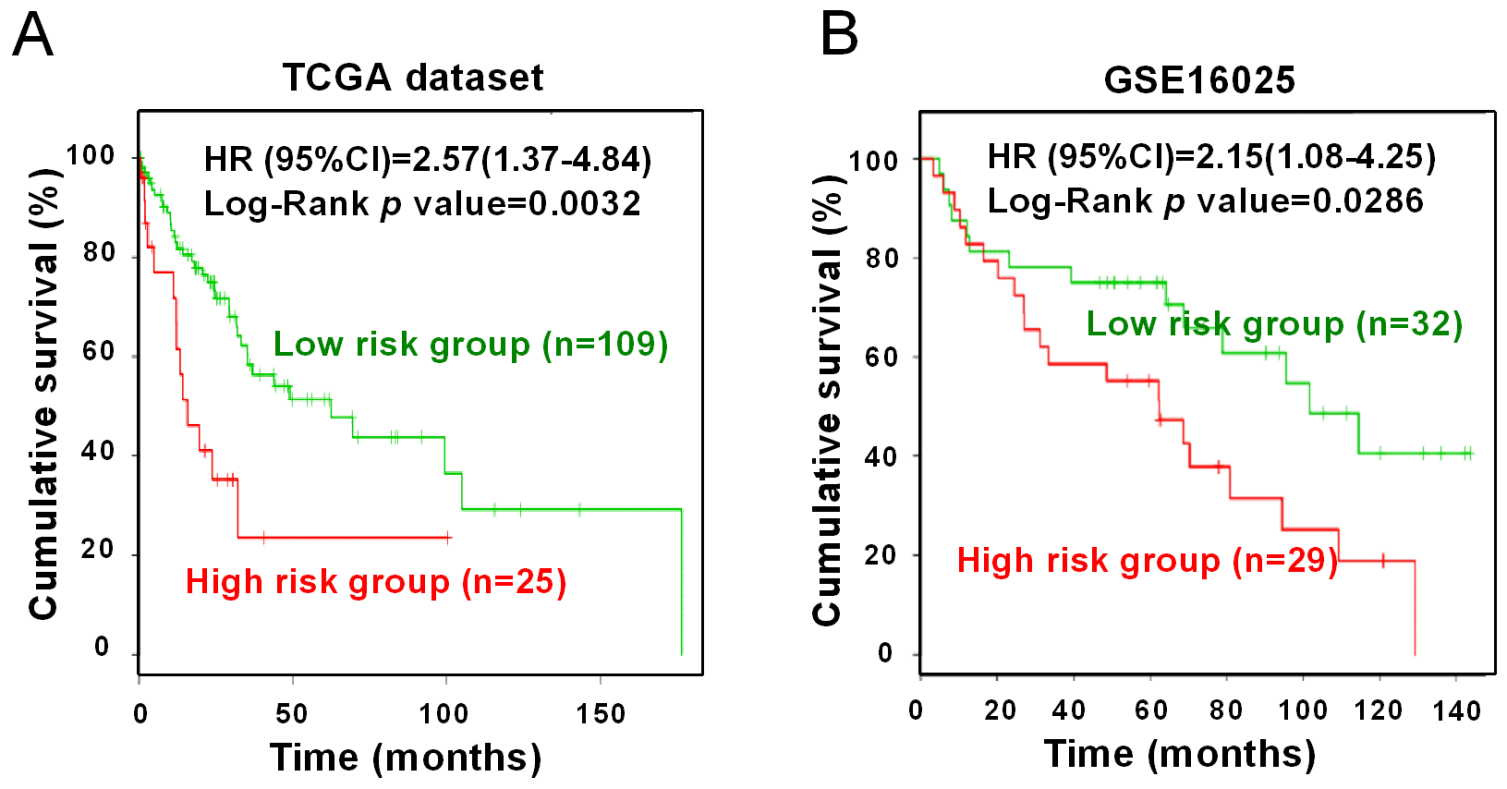
Survival analysis of three miRNAs Kaplan-Meier survival curves were conducted to compare overall survival of SCC patients in high or low risk groups. **(A)** TCGA; **(B)** GSE16025. Green: low risk group. Red: high risk group.

Kaplan-Meier curve was conducted to analyze the association between three miRNAs (miR-203, miR-205 and miR-375) expression and overall survival in SCC patients. To reduce inter-lab variability, prognostic data were obtained from two independent datasets based on sequencing (TCGA-LUSC dataset, n=134) and microarray platforms (GSE16025 dataset, n=61), respectively. By using the SurvMicro web tool, we compared the overall survival between the SCC patients with high risk and low risk groups, according to their expression levels of three miRNAs. As shown in Figure 8, combination of miR-203, miR-205 and miR-375 was significantly associated with overall survival of SCC in both two datasets, with Log-Rank *p*-values being 0.0032 and 0.0286, respectively. The Cox regression model suggested that SCC patients in high risk groups showed shorter overall survival time, compared with those in low risk groups. The corresponding Hazard ratio (HR) values were 2.57 (95% confidence interval (CI), 1.37-4.84) and 2.15 (95%CI, 1.08-4.25) in TCGA and GSE16025 datasets, respectively, suggesting that three-miRNA signature was related to the overall survival in SCC patients.

## DISCUSSION

Lung cancer is initiated by various genetic alterations, and investigation of cancer genome landscapes have a profound effect on clinical practice [26]. Recently, high-throughput methods have been widely used to screen thousands of genes simultaneously which makes genomic study much more efficiently [27]. Bioinformatic analysis based on high-throughput data will definitely provide more evidence for understanding molecular mechanisms of carcinogenesis. Here, we employed three miRNA expression profiles (GSE19945, GSE25508 and GSE51853) to search for the key miRNAs as candidate clinical biomarkers in different histological subtypes of NSCLC. After the comprehensive analysis, miR-375, miR-203 and miR-205 were found to distinguish SCC from other subtypes of NSCLC. Moreover, function prediction and coexpression analysis suggested that above three miRNAs might affect cancer progression and Hippo signaling pathway. Finally, the prognostic values of three-miRNA signature were confirmed by two independent datasets.

Recent studies showed the clinical features varied among different NSCLC subtypes [29, 30]. SCC, accounting for some 30% of lung cancer cases, is more closely associated with tobacco smoking than ADC/LCLC [26, 28, 29]. Most SCCs are centrally located and tend to originate from proximal bronchi whereas ADC/LCLC are often peripheral [30]. ADC arises most frequently from bronchial mucosal glands and are highly vascular [31]. And the least common subtype, LCLC was highly malignant, one subtype of which has neuroendocrine properties [30]. ADC/LCLC was easier and earlier to metastasize than SCC, the latter always grows slowly and are more likely to remain in the central area of lung [32, 33]. Furthermore, the responses to targeted therapy are also different in patients with different histological subtypes [34, 35]. Therefore, exploration the underlying molecular changes among different histological subtypes might provide evidence for understanding mechanisms of NSCLC. In this study, we compared the miRNA expression profiles of SCC/ADC/LCLC samples with normal lung samples using three GEO datasets. From ADC vs. normal, there were two miRNAs significantly up-regulated (miR-210 and miR-130b) and none down-regulated. And from SCC vs. normal, 3 miRNAs (miR-205, miR-210 and miR-130b) were significantly up-regulated and 2 (miR-486 and miR-30a) were significantly down-regulated. As for LCLC vs. normal, 5 (miR-30a*, miR-30a, miR-497, miR-144 and miR-145) were significantly down-regulated and none up-regulated. Moreover, we compared different subtypes of NSCLC (SCC vs. ADC, SCC vs. LCLC and ADC vs LCLC) using the similar method. Interestingly, we found miR-203, miR-205 and miR-375 distinguished SCC from other NSCLC subtypes, consisting with previous studies [36-38]. Moreover, we also confirmed that the signature of three-above mentioned miRNAs was also associated with overall survival of SCC patients. Canueto et al. reported the prognostic values of miR-205 and miR-203 in cutaneous squamous cell carcinoma [39]. Additionally, miR-375 was also found to be negatively associated with clinical outcomes in NSCLC [40, 41]. Therefore, our above findings provide evidence for investigating mechanisms and exploring therapeutic target for NSCLC, especially for SCC patients.

In order to further explore the function of three identified miRNAs, pathway enrichment analysis of their target genes was performed. Hippo signaling pathway was the most predicted pathway affected by miR-203, miR-205 and miR-375. Hippo signaling pathway was reported to be closely related to the occurrence of multiple malignancies, including NSCLC [42-44]. Therefore, we further performed coexpression analysis of three miRNAs and Hippo signaling pathway related genes. Four hub genes (*TP63*, *RERE*, *TJP1* and *YWHAE*) were identified as the pivotal genes that might be affected by three miRNAs. Furthermore, our validation data confirmed the association between miRNAs and three Hippo signaling pathway related genes (*TP63*, *TJP1* and *YWHAE*). *TP63* (tumor protein p63), a member of p53 family, was considered as one of “squamous markers” [45], and its copy number variation was reported to be associated with SCC [46]. *TJP1* (tight junction protein 1), also known as *ZO-1* (Zonula Occludens-1), participated in cell-cell adhesion and is reported to be associated with skin SCC [47]. Recent studies suggested that *TJP1* might be a useful prognostic predictor of NSCLC [48, 49]. *YWHAE*, a gene encoding 14-3-3 protein, was found to involve in carcinogenesis and progression of SCC [50]. Taken together, both our findings and previous reports suggested the critical roles of three miRNAs (miR-203, miR-205 and miR-375) in regulating Hippo signaling pathway. Therefore, investigation the underlying interaction between above mentioned miRNA and genes will be of particular interest to study the molecular mechanisms of NSCLC.

In conclusion, we identified differentially expressed miRNAs and their possible target genes involved in different histological subtypes of NSCLC. The novel findings in the present study were that miR-203, miR-205 and miR-375 could distinguish SCC from other subtypes of NSCLC, and were associated with overall survival in SCC patients. Hippo signaling pathway might be affected by above-mentioned three miRNAs. Our results provide evidence to better understand the molecular changes underlying distinctive biological properties of different NSCLC subtypes.

## MATERIALS AND METHODS

### Microarray datasets

The miRNA datasets were searched from the GEO database (https://www.ncbi.nlm.nih.gov/geo/). The search strategy was (“lung” OR “pulmonary” OR “respiratory” OR “bronchi” [Mesh]) AND (“cancer” OR “carcinoma” OR “tumor” OR neoplas* OR malignan* OR “squamous Cell Carcinoma” OR “adenocarcinoma” OR “large cell lung cancer” [Mesh]) AND (MicroRNA OR miRNA [Mesh]). Finally, three datasets (GSE19945, GSE25508 and GSE51853) were selected for further analysis. These three datasets contained the histological information of NSLC (ADC, SCC and LCLC).

GSE19945 was conducted through GPL9948 platform (Agilent Human 0.6K miRNA Microarray G4471) and consisted of 4 ADC, 5 SCC, 11 LCLC and 8 normal lung tissue samples. Meanwhile, GSE51853, based on GPL6480 platform (Agilent-014850 Whole Human Genome Microarray 4x44K G4112F), consisted of 76 ADC, 29 SCC, 17 LCLC and 5 normal lung tissue samples [18]. GSE25508 was conducted by GPL7731 platform (Agilent-019118 Human miRNA Microarray 2.0 G4470B) to explore miRNA expression panel with or without asbestos exposure [19]. From GSE25508 dataset, miRNA profiling data of 10 ADC, 8 SCC, 4 LCLC and 22 non-cancer lung tissue samples were extracted for analysis.

### Identification of DEmiRNAs

DEmiRNAs between different groups (cancer vs normal, or comparison among different histological subtypes) were screened based on three GSE datasets, respectively. Only fold change (FC)>=1.5 and *p*-value for *t*-test <0.05 was considered statistically significant. The heatmap of DEmiRNAs was conducted by a web-based tool, Morpheus (https://software.broadinstitute.org/morpheus/). Common DEmiRNAs among three GSE datasets was conducted by Venny 2.1.0 (http://bioinfogp.cnb.csic.es/tools/venny/). Moreover, we carried out a comprehensive meta-analysis of the expression of the miRNA screened above through a RRA method to identify DEmiRNAs [17]. This approach requires ranked miRNA lists and the total probe number of each miRNome profiling study. MiRNAs were re-ranked by corrected *p*-value.

### Prediction of target genes

Target genes of the DEmiRNAs was predicted by a web tool, TargetScanHuman 7.1 (http://www.targetscan.org/vert_71/). The cumulative weighted context++ score <-0.4 (-0.2 for miR-375) was adopted as the cut-off value. These genes were intended for pathway enrichment. GSE51852, another subseries of GSE51855, was a gene expression profile based on the same sample as GSE51853. The interaction between the DEmiRNAs and the significantly genes was mapped in Cytoscape [20].

### Pathway enrichment analysis

Gene Ontology (GO) and KEGG pathway enrichment was performed by Database for Annotation, Visualization and Integrated Discovery (DAVID) (https://david-d.ncifcrf.gov/summary.jsp), a web tool for gene functional annotation [21]. Identifier of official gene symbol and homo sapiens were selected.

### Cell culture and transfection

The HCC cell line, SMMC-7721, used in this study were obtained from the Liver Cancer Institute, Fudan University (Shanghai, China), and maintained in DMEM containing 10% fetal bovine serum at 37°C with 5% CO_2_. The miRNA mimics were synthesized by GenePharma (Shanghai, China). The transfections of miR-375 and miR-203 mimics were performed using Lipofectamine 2000 (Invitrogen, Carlsbad, CA, USA) according to the procedure recommended by the manufacturer.

### Reverse transcription-polymerase chain reaction (RT-PCR)

Total RNA was isolated from the different cell groups using the RNAfast200 Total RNA Extract Kit (Fastgene, Shanghai, China), and 2 μg RNA was reverse transcribed to cDNA by the RevertAid™ First Strand cDNA Synthesis Kit (Fermentas, MBI, Lithuania). Quantitative RT-PCR was carried out using the SYBR^®^ PrimeScriptTM miRNA RT-PCR Kit and SYBR^®^ Premix Ex TaqTM (TaKaRa Biotechnology, Dalian, China). The Fold change of gene expression was calculated based on the threshold cycle (Ct) as relative to β-actin using a 2-Δ(ΔCt) method. The sequences of PCR primers for target genes are listed as followings. *TP63*: forward primer, 5’-GGACCAGCAGATTCAGAACGG-3’; reverse primer, 5’-AGGACACG TCGAAACTGTGC-3’; *TJP1*: forward primer, 5’-CAACATACAGTGACGCTTCACA-3’; reverse primer, 5’- CACTATTGACGTTTCCCCACTC-3’; *YWHAE*: forward primer, 5’- GATTCGGGAATATCGGCAAATGG-3’; reverse primer, 5’-GCTGGAATGAGGTGTTTG TCC-3’. All primer pairs were synthesized by TaKaRa.

### Survival analysis

The prognostic value of DEmiRNAs was evaluated by SurvMicro (http://bioinformatica.mty.itesm.mx:8080/Biomatec/Survmicro.jsp) [22]. Kaplan-Meier curves were conducted based on TCGA-LUSC and GSE16025 datasets. MiRNA profile of TCGA-LUSC dataset was sequenced using Illumina Genoma Analyzer IIx Equipment. GSE16025 dataset contained 61 SCC samples and 10 matched normal lung samples and profiled on MirVana miRNA Bioarrays (version 2, Ambio). HR and 95% CI of the association between miRNAs and overall survival was estimated using a Cox proportional hazards analysis. Statistical assessment was performed using the Log Rank test.

### Statistical analysis

Statistical analyses were performed using STATA software, version 12.0. The paired student *t*-test was used to compare the differences in gene expression between control and miRNA mimics -transfected HCC cells. Pearson correlation was applied to evaluate the expression correlation between the DEmiRNAs and the selected genes. *p*-value less than 0.05 was considered statistically different.

## Abbreviations

ADC: lung adenocarcinoma
CI: confidence interval
Ct: threshold cycle
DAVID: Database for Annotation, Visualization and Integrated Discovery
DEmiRNA: differentially expressed miRNA
FC: fold change
GO: Gene Ontology
GEO: Gene Expression Omnibus
HR: hazard ratio
KEGG: Kyoto Encyclopedia of Genes and Genomes
LCLC: large cell lung cancer
NSCLC: non-small cell lung cancer
miRNA: microRNAs
NCBI: National Center for Biotechnology Information
RRA: robust rank aggregation
SCC: lung squamous cell carcinoma
